# Environmental Toxicants in the Hispanic Community Epigenetically Contributing to Preeclampsia

**DOI:** 10.1007/s12012-025-10049-9

**Published:** 2025-07-31

**Authors:** Lauren Rae Gladwell, Laura Packer, Jhanvi Karthik, James Tinwah Kwong, Raina Hummel, Yuting Jia, Samiran Sinha, Theresa Morris, Robin Page, Mahua Choudhury

**Affiliations:** 1 Department of Pharmaceutical Sciences, Texas A&M Irma Lerma Rangel College of Pharmacy, College Station, TX USA; 2 Department of Statistics, Texas A&M University, College Station, TX USA; 3 Department of Sociology, Texas A&M University, College Station, TX USA; 4 College of Nursing, Texas A&M University, College Station, TX USA

**Keywords:** Epigenetics, Hispanic, Phthalates, Air pollution, Metals, Preeclampsia

## Abstract

Preeclampsia is a multisystemic cardiovascular pregnancy complication that endangers the lives of both mother and child. Its prevalence disproportionately burdens women of different races and ethnicities nationwide. Specifically, Hispanic women experience higher rates of preeclampsia compared to Non-Hispanic White women; however, the explanation for this phenomenon is still elusive. To decipher these disparate rates, environmental factors that the Hispanic community is excessively exposed to may need to be considered. Environmental toxicants such as particulate matter, heavy metals, phthalates, as well as microplastics and nanoplastics are ubiquitous within the Hispanic environment. From places of employment to in the home, the role of chronic toxicant exposure in Hispanic women may begin to explain the gap in the prevalence of preeclampsia development. To understand the relationship between toxicant exposure and preeclampsia, we explored epigenetic concepts. With the capacity to respond to the environment in a heritable and reversible manner, epigenetics changes the expression of genes and proteins while leaving the DNA sequence intact. Epigenetic modifications can be dysregulated upon toxicant exposure and can potentially lead to the development of preeclampsia. Here, in this review, we propose the potential epigenetic links through which Hispanic women’s disproportionate environmental exposure to toxicants can be conducive to preeclampsia development.

**Graphical Abstract:**

Hispanic women’s environmental exposure to toxicants may induce epigenetic dysregulations within the placenta, leading to preeclampsia. *Wordart.com* and BioRender were used to generate the figure.

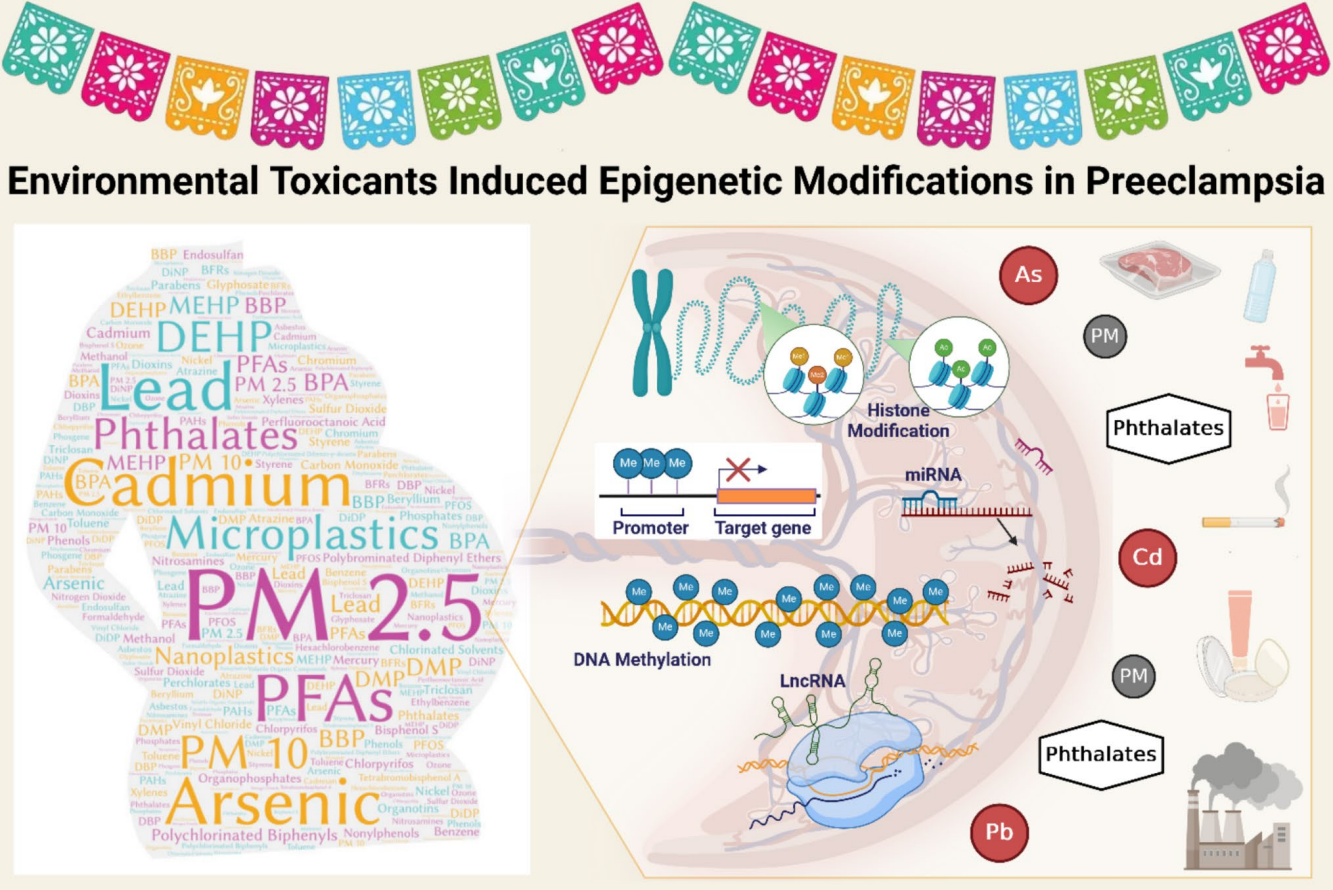

## Introduction

Preeclampsia is one of the leading contributors to maternal mortality and morbidity regardless of country [1, 2]. Impacting approximately 2–8% of all pregnancies globally, preeclampsia results in the deaths of 76,000 mothers and 500,000 infants each year [3]. These rates only become greater in low-income and developing areas [4, 5]. Preeclampsia does not affect women of all races and ethnicities equally [6–8]. Hispanic women are found to have higher rates of preeclampsia and preeclampsia-related mortality compared to Non-Hispanic White women [9–11]. Despite the Hispanic population being the fastest growing population in the United States [12], they are still an understudied and underrepresented group [13–15]. Hispanic women are also faced with an increased prevalence of pregnancy complication risk factors such as cardiovascular disease (CVD) and type 2 diabetes mellitus (T2D) [9, 16]. In the United States between 2007 and 2018, the prevalence for hypertensive disorders of pregnancy (HDP) in Hispanic women rose from 2.8% to 5.9%, resulting in a 2.23 odds ratio [17]. A similar pattern for T2D was found as the prevalence in Hispanic women elevated from 4.6% to 8.3% with a 1.88 odds ratio [17]. Notably, the prevalence of HDP and T2D in women of all races and ethnicities investigated was identified to only increase over time, but the biggest rises were in Hispanic women [17]. Although there is no known cause for preeclampsia, recent evidence suggests the role of environmental toxicants such as air pollutants, heavy metals, forever chemicals, and plastics in this scenario [18–22]. Specifically, current research has revealed systemic inequalities in the exposure to environmental pollution in populations of color compared to their Non-Hispanic White counterparts [23–25]. This poses the question of how do these differences in toxicant exposure of Hispanic women contribute to the disproportionate rates of preeclampsia. The recent field of epigenetics proposes a potential explanation by linking environmental exposures to preeclampsia [26]. In this review, we will describe a path in which specifically Hispanic women are exposed to environmental toxicants that can potentially induce epigenetic dysregulations leading to preeclampsia.

## Preeclampsia

Preeclampsia is characterized by the presence of hypertension and the onset of at least one associated pregnancy complication [27]. Preeclampsia is typically diagnosed after the 20th week of gestation and is a multisystem cardiovascular disorder [28, 29]. The most common associated complication is protein in the urine, referred to as proteinuria; although, in its absence, cardiorespiratory dysfunction, kidney or liver injury, as well as other related symptoms are considered [29, 30]. If unattenuated, preeclampsia can progress to induce AFLP (acute fatty liver of pregnancy), HELLP (hemolysis, elevated liver enzymes, and low platelets) syndrome, placental abruption, stroke, and death [31–35]. The only currently known treatment is to deliver the fetus; however, this may not always be an option [36]. Often, preeclampsia induces higher rates of stillbirths, pre-term births, and early neonatal deaths [37–40]. Those born from preeclamptic mothers are also more likely to be born with low birth weight, small for gestational age, or large for gestational age [41, 42]. The ramifications of preeclampsia persist after birth as well. Mothers diagnosed with preeclampsia are predisposed to increased CVD morbidity and mortality [43–47], end stage renal disease [48–50], cognitive impairment or dementia [51], and T2D [52, 53]. The babies born to mothers with preeclampsia can also face life-long negative outcomes. They are at a higher risk for neurological disorders such as autism spectrum disorder, attention-deficit/hyperactivity disorder, cerebral palsy, epilepsy, intellectual disability, and vision or hearing loss [54, 55]. Further, babies from mothers that experienced more severe or earlier onset of preeclamptic complications carry an increased risk for stroke or developing hypertension themselves, respectively [56–58]. As Hispanic mothers face the adversity of disproportionate rates of preeclampsia, looking at this disorder from a Hispanic-focused lens may offer promise.

## Environment of Hispanic Communities

**Fig. 1:**
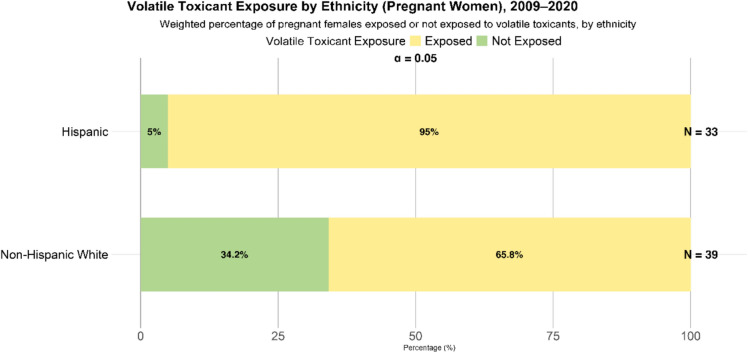
Volatile toxicant exposure by ethnicity and age, National Health and Nutrition Examination Survey (NHANES). Weighted percentage of pregnant women (2009–2020) reporting exposure versus no exposure to volatile toxicants (sourcing from mothballs, toilet deodorizers, nail polish fumes, dry cleaning solvents, cooking with natural gas, and refueling vehicles), grouped by Hispanic or Non-Hispanic White ethnicity. The survey sample sizes (N) appear to the right of each bar. Yellow segments represent the proportion “Exposed,” while green segments represent “Not Exposed.” The significance level for comparing exposure rates between the two groups is α = 0.05 [63]

There are 16.8 million Hispanic people living in counties that have severely poor air quality with unhealthy levels of annual and daily particulate matter (PM) exposure as well as ozone exposure [59]. Along with air quality, public drinking water from residential areas characteristically populated by Hispanics was found to have significantly increased levels of toxic uranium (U) and arsenic (As) [60]. The jobs taken on by the Hispanic population can pose a factor as well. While only making up 19% of the total United States workforce in 2022, Hispanic men and women dominated employment as construction workers, maids and housekeeping cleaners, carpenters, landscaping and groundskeeping workers, painters, and paperhangers [61]. Hazards of these occupations such as exposure to toxicants can be especially harmful to pregnant women. As Hispanic women are evidenced to continue to work while pregnant, this highlights how much more susceptible they can be to preeclampsia due to toxicant exposure [62]. According to the Centers for Disease Control and Prevention, pregnant Hispanic women were found to have significantly higher exposure rates to volatile toxicants at 95%, whereas Non-Hispanic White women were at 65.8% (Fig. 1) [63]. Volatile toxicant exposures were assessed through the use of mothballs, toilet deodorizers, nail polish fumes, dry cleaning solvents, cooking with natural gas, and refueling vehicles [63]. It is important to note that a limitation of this comparison is the small sample size as it may not wholly reflect the entire population. Thus, more epidemiological exposure studies are required to clearly establish the association of toxicant exposure among different racial and ethnic populations. This only begins to give a glimpse into the potential exposure rates and different routes that pregnant Hispanic women may be exposed to toxicants. From the air [64], water [65], to their workplaces [66, 67], the environmental disparities that Hispanic women face can play a prominent role in their health and during pregnancy.

## Non-communicable Diseases in the Hispanic Community

It is well known that obesity, T2D, and CVD are risk factors for preeclampsia [68, 69]. When compared to Non-Hispanic White women, Hispanic women exhibit higher rates for these risk factors before and during pregnancy [68, 70]. Evidence has also pointed to the Hispanic population being genetically predisposed to these health issues as well. Various studies with Native Mexicans and Mexican Americans found single-nucleotide polymorphisms (SNPs) associated with increased rates of obesity, waist circumference, triglyceride levels, and insulin resistance [71, 72]. For example, a cross-population allele screening between people of Mexican and Finnish ancestries to assess potential genes contributing to Amerindian (e.g., Mexican Americans) risk of CVD development revealed several variants in lipid-related loci [72]. These included lipoprotein lipase (LPL), salt-inducible kinase 3 (SIK3), and receptor-related orphan receptor alpha (RORA), but through an oral fat tolerance test they demonstrated that SIK3 variant carriers had more pronounced pre- and postprandial serum triglyceride levels than non-carriers [72]. Notably, the authors suggest that methylation of LPL, an epigenetic mechanism, may play more of a role as the variant can affect the site for DNA methylation within the gene [72]. A separate study evaluating the role of SNPs in interleukin 6 (IL-6) in Mexican Americans from South Texas identified the rs1800796 variant to be correlated with insulin resistance, larger waist circumference, increased high-sensitive C-reactive protein, and reduced IL-6 levels [71]. Interestingly, this IL-6 variant was also found among European and Asian populations yielding similar associations with insulin resistance and obesity [73–75]. This presents the notion that despite containing a similar variant, the extent of the SNP effect differs per population. The diverse ancestry of the Hispanic population adds another layer of complexity that makes attributing metabolic predispositions to solely SNPs difficult [76]. Specifically, the varied proportions of Native American, European, and African ancestries within Colombian, Mexican, Puerto Rican, and Peruvian populations correlated with differing genetic predispositions in health pertaining to immunity, metabolism, and disease susceptibility [76]. It also has recently been described that epigenetic mechanisms can influence the manifestation of SNPs [77–79]. These findings present that epigenetic changes driven by differences in environments may contribute more compared to only genetics as previously conceptualized. Overall, this demonstrates that these genetic predispositions alone cannot explain the disproportionate increased risk of Hispanic women to contributing factors for preeclampsia. To bridge the gap between these environmental exposures, genetic predispositions, and preeclampsia, epigenetics offers new perspectives [26, 80].

## Principle Epigenetic Marks

Epigenetics is the field of study that describes how environmental factors can impact gene expression without imposing any changes to the DNA sequence itself [81]. Epigenetic modifications are both heritable and reversible. There are 3 key epigenetic modifications that mediate these changes to gene expression: DNA methylation, histone modification, and noncoding RNAs. DNA methylation is the addition of a methyl group, often on cytosines that come before guanine (CpG) sites, on the DNA [82]. Increased DNA methylation typically acts as a repressive mark to silence genes, whereas decreased methylation enables the transcription of the gene [83]. There are also circumstances where increased methylation can promote gene expression, such as if it is localized to a gene’s promoter region [83]. The addition and removal of these methyl groups is mainly conducted by the two opposing groups of DNA methyltransferases (DNMTs) and ten–eleven translocation (TET) proteins, respectively [82]. Of note, 5-hydroxymethyl-cytosine (5hmC) that is produced from DNA demethylation is emerging as a potential epigenetic mark itself, yet its regulatory capacity is still being discovered [84].

The next prevalent epigenetic modification is histone modification. Histones are proteins that help condense and wrap large sections of DNA into smaller units called chromatin [85]. These histones have tails that can be modified post-translationally through the addition or removal of different groups to alter the charges of the tails [86]. This can change the binding affinity of the histone to the chromatin, modulating DNA accessibility, and subsequently gene expression [86]. Major histone modifications include methylation and acetylation that often occur at the lysine residues of histone tails [85]. Similar to DNA methylation, the positioning of histone modifications such as along promoter regions or within the gene body can also contribute to their regulatory nature [85]. Multiple methyl groups can be added to a single residue and thus the quantity of methyl groups can indicate how genes are expressed or suppressed. For example, increased methylation at histone 3 lysine 4 (H3K4) is considered an active mark, whereas increased methylation of H3K9 is recognized as a repressive mark [85, 87]. Conversely, histone acetylation consists of acetyl groups (ac) being added to positively charged lysine residues to prevent histone tails from tightly binding to the negatively charged DNA, thus permitting gene expression [88]. This innately affords the epigenome the flexibility such as to switch from repression through H3K9 trimethylation (me3) to transcription via H3K9ac. These reversible qualities of histone modifications are provided by histone modifying enzymes. Histone acetylation is directed by two contrasting groups of enzymes—histone acetyltransferases (HATs) and histone deacetylases (HDACs) [88]. HATs are responsible for adding acetyl groups to these lysine residues, while HDACs will remove them [85]. Contrastingly, histone methylation is primarily dictated by methyltransferases (KMTs) for addition and demethylases (KDMs) for removal of methyl groups [89].

The last major epigenetic player is noncoding RNAs. There are two predominant epigenetic categories of noncoding RNAs that are separated based on their sizes: microRNAs (miRNAs) and long noncoding RNAs (lncRNAs). LncRNAs are over 200 nucleotides in length, whereas miRNAs are approximately 22 nucleotides long [90, 91]. miRNAs are well recognized to bind to complementary sequences along DNA and messenger RNA (mRNA) to regulate gene expression [90]. Conversely, lncRNAs have a multitude of functions including those of miRNAs as well as directing, interacting, and sequestering other epigenetic modulators to alter gene expression [92]. Interestingly, altered levels of miRNAs are proposed to be indicators for different diseases or conditions [93–95]. A beneficial quality of these noncoding RNAs, specifically miRNAs, is their ability to be detectable in several bodily fluids such as blood, serum, and saliva [96, 97]. This presents several opportunities from serving as biomarkers for disease development to being therapeutic targets themselves [98, 99]. Recently, our lab patented a panel of epigenetic modifications, including miRNAs, that can serve as biomarkers for preeclampsia [100]. This begins to evidence the potential of approaching preeclampsia from an epigenetic perspective. Further research at a racial- and ethnic-specific level is required to fully leverage the power of epigenetics in preeclampsia. By investigating the environmental toxins to which Hispanic women are mostly exposed, we can begin to understand prospective underlying epigenetic mechanisms that may contribute to the racial and ethnic disparities of preeclampsia.

## Air Pollutants

An inescapable route for toxicant exposure is via the air. However, the quality of air that different populations are exposed to may not always be equal. Exposure can occur at places of employment, schools, community centers, and even at home [101, 102]. Thus, we reviewed where the Hispanic population might be more heavily exposed. Containing a mixture of solid and aerosolized particles, particulate matter (PM) is a prevalent form of air pollution that is typically absorbed through inhalation [103]. There are two categories of PM– PM_10_ that has a diameter between 2.5 and 10 µm and PM_2.5_ which has a diameter smaller than 2.5 µm [103]. There is a recent proposal that alongside negatively impacting the respiratory system, PM exposure can also potentially contribute to the development and worsening of CVD [104–107]. In particular, a meta-analysis investigating the effects of short-term and long-term exposure to air pollutants revealed PM to be significantly correlated with hypertension at both timeframes [108]. In healthy pregnant women with no prior incidence of hypertension or diabetes, PM_2.5_ exposure was correlated with a rise of maternal blood pressure [109]. Additionally, a Spanish case–control analysis revealed that PM_2.5_ exposure was positively associated with the development of preeclampsia [110]. PM_2.5_ exposure of Hispanic women in California, specifically those belonging to low and middle-income communities, was also strongly correlated with a higher risk of development of preeclampsia [111]. Further, Hispanic pregnant women were found to have poorer pregnancy outcomes compared to Non-Hispanic White women due in part to exposure to PM_2.5_ [112]. Highly frequent indoor modes of PM_2.5_ exposure for Hispanic women are via smoking, candle/incense burning, cooking, and use of heating units [113–115]. In a study examining the indoor levels of PM_2.5_ and its sources in a low-income community, activities such as cooking, smoking, and use of the range hood were significantly correlated with higher concentrations of PM_2.5_ in the home [113]. The study population was mainly composed of Hispanic women, and the authors noted indoor PM_2.5_ exposure can have implications in racial, ethnic, and socioeconomic disparities [113]. Xu and colleagues identified similar contributors of indoor PM_2.5_ among a predominantly Hispanic cohort of pregnant women, the Maternal and Developmental Risks from Environmental and Social Stressors (MADRES) cohort [116]. This cohort was previously established with the objective of improving maternal and childhood obesity in lower-income communities within Los Angeles [117]. Xu et al. also provided a unique source of PM_2.5_ exposure from airborne sea salt from the Pacific Ocean [116]. Interestingly, a separate study by Niu et al. analyzing the same MADRES cohort revealed an association between 1st trimester PM_2.5_ exposure and an increased risk for gestational hypertension but not with preeclampsia [118]. The authors note a possible reason for this is due to their limited scope of the first half of pregnancy (e.g., gestational weeks 1–20), when preeclampsia most often gets diagnosed in the latter half of pregnancy (e.g., gestational weeks 21–40) [118]. The above evidence indicates that the airborne toxicants can be one of the plausible causes for the development of preeclampsia in Hispanic women.

One possible mechanism through which PM exposure can contribute to preeclampsia is through the downregulation of the pivotal DNA methylation mediator DNMT3b [119]. Responsible for de novo methylation, DNMT3b is an established vital epigenetic regulator during embryonic development [120]. The deletion of DMNT3b in a knockout mouse model led to a reduction in placental size through the stunting of the labyrinth zone, leading to the lethality of the embryo [121]. Several genes that are distinctly increased in healthy placentas such as CEA cell adhesion molecule 14 (CEACAM14), fibronectin 1 (FN1), and cathepsin 3 (CTS3) were markedly downregulated in the DMNT3b knockout model [121]. As severe impairment of placental development reduces capacity for nutrient and gas exchange with the fetus and is a hallmark of preeclampsia, this begins to elucidate how PM exposure can contribute to its development (Table 1) [122, 123].

**Table 1:** Epigenetic dysregulations and their downstream effects caused by Particulate Matter_2.5_

Potential sources	Air pollutant(s)	Epigenetic modifier(s)/ modification(s)	Downstream target(s)	Functional endpoint(s)/mechanistic connection
Smoking, candle/incense burning, cooking, airborne sea salt as well as use of heating units and range hoods [113–115, 143]	PM_2.5_	DNMT3b [121]	CEACAM14, FN1, CTS3 [121]	Impaired placental development [121]
	PM_2.5_	H3K9ac [124]	GATA4, MEF2C [124, 128]	Fetal growth restriction [128]
	PM_2.5_	p300/CBP- HIF-1α [134, 136]	VEGF, sFLT1 [135]	Placental dysfunction via HIF-1α Signaling Pathway [135, 136]

As fetal exposure to preeclampsia is proposed to leave long-lasting cardiovascular effects on the offspring, it is plausible that the epigenetic determinants for the development of CVD later in life can have been instigated in utero. For instance, the offspring of pregnant mice exposed to PM_2.5_ throughout gestation later developed cardiac hypertrophy as adults [124]. This was proposed to be mediated by the increased acetylation by HATs p300 and CBP at H3K9 of Gata binding protein 4 (GATA4) and myocyte enhancer factor 2C (MEF2c) promoter regions, leading to their upregulation [124]. Interestingly, in the cord blood of preeclamptic pregnancies, it was observed that the GATA4 binding motif region was significantly upregulated, potentially indicating higher transcriptional activity [125]. Additionally, MEF2c is typically lowly expressed in trophoblast cell lines such as HTR8/SVneo and BeWo as well as in the first trimester of pregnancy [126, 127]. It is further described that female offspring exposed to lipopolysaccharide (LPS) in utero later presented with increased MEF2c expression in their hearts and fetal growth restriction during their pregnancies [128]. This evidence can possibly link how early epigenetic dysregulations obtained in utero due to toxicant exposure can be conducive to both pregnancy disorders and adulthood CVD development.

In addition to regulating the expression of GATA4 and MEF2c, p300 regulates a myriad of other functions within the placenta [129–133]. Its regulatory function can be manipulated by different transcriptional factors such as hypoxia-inducible factor-1α (HIF-1α) to dysregulate the sensitive balance of acetylation [134]. It was determined that overexpression of HIF-1α in pregnant mice led to a preeclamptic phenotype including increased blood pressure and proteinuria [135]. Correspondingly, when human umbilical vein endothelial cells (HUVECs) were exposed to PM_2.5,_ there was a concomitant dose-dependent increase in HIF-1α expression [136]. The hypoxic response of HIF-1α and p300/CBP is essential to angiogenesis, but in the presence of toxicant exposure it can be dysregulated. With crucial downstream genes such as vascular endothelial growth factor (VEGF) and its receptor soluble vascular endothelial growth factor receptor-1 (sFLT-1), this overcompensated response can lead to preeclampsia [135, 137]. A combined increase in sFLT-1 and decrease in VEGF expression is a common indicator for preeclampsia and is recognized to disrupt placental function as well as structure [138–142]. It is plausible that PM exposure exacerbates hypoxia and subsequently HIF-1α to excessively commandeer p300/CBP and dysregulate angiogenesis. Thus, this displays another possible mechanism through which exposure to PM, that Hispanic women are highly exposed to, can be conducive of preeclampsia development. As PM can be composed of a variety of both biological and chemical components, it is necessary to also understand how the chemicals commonly found within PM may independently contribute to preeclampsia.

## Heavy Metals

Exposure to a group of toxic chemicals known as heavy metals is revealed to be a risk factor in the development of preeclampsia [144–146]. These metals include cadmium (Cd), arsenic (As), and lead (Pb) whose exposure routes can be through contaminated air, water, food, and surface contact [147]. Heavy metal exposures are toxic to all women, although the frequency of exposure among different racial and ethnic groups may vary. Mijal et al. revealed a 1.77 greater odds of high levels of blood Cd among pregnant Mexican American women who never smoked than their Non-Hispanic White counterparts [148]. Cd is a key toxicant produced by tobacco smoke [149]; thus, their findings were unexpected as the Mexican American women had overall lower Cd levels, due to less prevalence of smokers, than Non-Hispanic White women [148]. The authors also noted elevated levels of Pb within the pregnant Mexican American women with the highest Cd levels, except they could not analyze the correlation due to limited statistical power [148]. In a separate study examining the PRogramming of Intergenerational Stress Mechanisms (PRISM) pregnancy cohort, Hispanic women were reported to have greater levels of Cd (141.5%), chromium (108.2%), Pb (59.9%), and antimony (38.3%) in their urine compared to Non-Hispanic White women [150]. Delving further, women belonging to lower socioeconomic neighborhoods were also associated with having increased urinary levels of these metals [150]. In a cross-sectional analysis of the MADRES cohort evaluating the levels of As in the urine, Farzan and colleagues identified a mean of 9.0 μg/L of total As across the Hispanic-dominated cohort [151]. These levels indicated only low to moderate As exposure within the cohort. We thus discussed below how the altered epigenetic regulations from exposure to heavy metals Cd, As, and Pb can possibly impact the development of preeclampsia in Hispanic women.

Cd is a thoroughly investigated heavy metal for its significance in preeclampsia [152–156], but the identification of disrupted epigenetic modifications facilitating this development is still in its infancy. In placental samples from preeclamptic patients, several miRNAs and genes belonging to the transforming growth factor-beta (TGF-β) pathway, responsible for migration of trophoblasts, were prominently dysregulated [157]. A portion of these miRNAs were notably associated with Cd exposure and recognized to target TGF-β related genes; thus, an in vitro placental model of Cd exposure revealed significantly dysregulated miRNAs alongside TGF-β genes [157]. Brooks et al. conducted a follow-up study investigating further into the physiological repercussions behind Cd-mediated preeclampsia development and reported diminished trophoblast migration upon Cd exposure [158]. They hypothesized that the impaired migration was due to the upregulated signaling of the TGF-β pathway facilitated by the downregulation of the TGF-β-suppressive miR-26a [158]. There is corroborating evidence highlighting the reduction of miR-26a with Cd exposure [159] and a placental role for miR-26a [160]; yet further clinical research is still required to fully elucidate its implication in preeclampsia.

Another heavy metal examined for its association with preeclampsia is As [161, 162]. Through a combinatorial transcriptomic, proteomic, and epigenomic approach, a network of epigenetic modulators and genes was determined to be altered with exposure to As during pregnancy [163]. HDAC4 in particular experienced increased methylation leading to decreased mRNA expression upon As exposure [163]. In a separate study, HDAC4 and forkhead box protein M1 (FOXM1) expression were described to be significantly reduced alongside a pronounced rise in miR-134-5p in placentas belonging to preeclamptic patients [164]. MiR-134 is recognized as a potential biomarker for preeclampsia and can impede the infiltration of trophoblasts [165]. Xu and colleagues proposed that HDAC4 negatively regulates miR-134-5p expression through deacetylation of promoter region H3K9 residues [164]. Upon HDAC4 knockdown, the abundant miR-134-5p sequesters FOXM1 to obstruct trophoblast invasion, proliferation, and migration [164]. This mechanism is strengthened by other observations of decreased levels of FOXM1, increased miR-134, and decreased HDAC4 in preeclampsia [166–169]. Cumulatively, this indicates the promotive role of As in preeclampsia development to which Hispanic women are disproportionately exposed.

Lastly, the heavy metal Pb also presents concern to fetal and maternal health [170] including preeclampsia [171]. A study conducted by Sen et al. reported differential levels of DNA 5hmC in cord blood belonging to Pb-exposed pregnant Mexican women through an HMeDIP-450 K assay they established in vitro using human embryonic stem cells [172].These alterations in 5hmC were located at promoter regions of Argonaute 2 (AGO2) a protein within the RNA-induced silencing complex (RISC) as well as essential mitochondrial genes responsible for ATP synthesis, sulfide catabolism, and reduction of oxidative radicals [172]. In preeclamptic placentas, AGO2 protein expression was found to be downregulated alongside prominent expression of miR-15b [173]. Expression of miR-15b was exhibited to alter AGO2 protein levels through the binding to conserved seed sequences within the AGO2 coding region and inhibit trophoblast invasion [173]. Using a miR-15b mimic, Yang et al. revealed increased sFLT secretion and proposed miR-15b to be promotive of preeclampsia pathogenesis via altering AGO2 to disturb trophoblast function (Table 2) [173]. Another study analyzing binding motifs related to intronic polyadenylation (IPA) events in preeclamptic patients described enrichment of binding motifs for AGO2 [174]. Polyadenylation is the addition of poly (A) tails to mRNA to assist in their stability before protein translation [175]. Zhang and associates also indicated an inverse relationship between IPA events and expression of genes within the VEGFA-VEGFR2 signaling pathway [174]. This suggests another role for AGO2 in preeclampsia via IPA of VEGF-related signaling pathways.

**Table 2:** Epigenetic dysregulations and their downstream effects caused by heavy metal exposure

Potential sources	Heavy metals	Epigenetic modifier(s)/ modification(s)	Downstream target(s)	Functional endpoint(s)/mechanistic connection
Contaminated air, water, food, surfaces, and tobacco smoke [147, 149, 151, 176]	Cadmium (Cd)	miR-26a [157–159]	TGF-β1, TGF-βR1/2, SMAD2, SMAD3 [157, 158]	Impaired trophoblast migration via TGF-β signaling pathway [157, 158]
	Arsenic (As)	HDAC4 [163, 164], H3K9ac [164]	miR-134, FOXM1 [164]	Obstructed trophoblastic invasion [164–166], proliferation [164], and migration [164, 167]
	Lead (Pb)	DNA 5hmC [172], miR-15b [173]	AGO2, sFLT [173, 174]	Inhibited trophoblast invasion [173] and dysregulated VEGFA-VEGFR2 signaling pathway [174]

## Phthalates and Microplastics

The unprecedented rise in plastic usage raises concern for the presence of their byproducts and the chemicals that stabilize them [177, 178]. Recent evidence indicates that Hispanic communities are disproportionately exposed to plasticizers known as phthalates due in part to socioeconomic factors [179, 180]. Phthalates are frequently found in food, cosmetics, medical devices, personal care products, feminine hygiene products, and dust in homes [181–184]. These are composed of diesters of phthalic acid and are used to make plastics more flexible and durable [185]. Exposure to phthalates is particularly concerning in the context of preeclampsia as they are proposed to disrupt endocrine signaling, metabolism, impair fertility, and induce adverse pregnancy complications, most notably, hypertension [180, 185–187]. Pregnant Hispanic women were found to have significantly higher levels of phthalate metabolites in their urine compared to Non-Hispanic White women who were correlated with increased incidence of pre-term birth [188]. Moreover, multiple phthalate metabolites in the urine from pregnant women were also revealed to be associated with placental developmental defects such as impaired microvasculature, placental stiffness, and calcification [189].

Our lab has previously evidenced the epigenetic repercussions of phthalate exposure both in vitro and in vivo in the fields of metabolic disease and CVD [190–198]. In human placental HTR-8/Svneo cells treated with mono-(2-ethylhexyl) phthalate (MEHP), we detected a dose-dependent elevation in hypoxia and oxidative stress as well as induction of mitochondrial dysfunction [199]. Alongside this, there was a dose-dependent increase in the expression of HIF-1α, miR-210-3p, and VEGFA [199]. We demonstrated a mechanism where MEHP induces the stability and activity of HIF-1α that in turn promotes the expression of miR-210-3p that induces oxidative stress and mitochondrial dysfunction (Table 3) [199]. Other studies we have conducted utilizing the same dosages of MEHP have revealed similar endpoints of cytotoxicity, induction of oxidative stress, and mitochondrial dysfunction in trophoblast cells as well [200, 201]. This can begin to describe how phthalate exposure can potentially lead to the dysfunction of trophoblasts through impaired invasion due to the incomplete remodeling of spinal arteries, thereby narrowing blood vessels and increasing blood pressure, leading to preeclampsia [202].

**Table 3:** Epigenetic dysregulations and their downstream effects caused by phthalates and MNPs

Potential sources	Phthalates, micro-, and nanoparticles (MNPs)	Epigenetic modifier(s)/modification(s)	Downstream target(s)	Functional endpoint(s)/mechanistic connection
Food, cosmetics, medical devices, personal care products, feminine hygiene products, and dust in homes [180–184]	MEHP	miR-210-3p, HIF-1α [199]	VEGFA, SDHB, NDUFB8, COX-IV [199]	Trophoblast dysfunction, generation of oxidative stress, and incomplete remodeling of spiral arteries via HIF-1α Signaling Pathway [199]
	Phthalate/MEHP	TET3 [213, 214], REST complex, Methylation and Hydroxy-methylation [214]	CRH [211, 212, 214]	Dysregulated blood flow and gestational period via NO/cGMP Signaling Pathway [210–213, 215]
Air, soil, food, plastic waste sites [220, 222]	MNP/Polystyrene	Unknown	Unknown	Unknown

Another potential route that phthalates can contribute to preeclampsia development is through inducing increased production of placental corticotropin-releasing hormone (CRH) [203–205]. Interestingly, there is also evidence of accompanying decreased expression in CRH receptor 1 in preeclampsia [206]. Coined as the placental clock, CRH’s main function is to regulate gestational length; however, it also regulates blood flow from the placenta to the fetus through the activation of the nitric oxide (NO)/cyclic guanosine monophosphate (cGMP) pathway [207, 208]. This frames a scenario where higher demand for blood flow, requiring activation of the NO/cGMP pathway, is impeded due to decreased expression of placental CRH receptors, resulting in increased CRH expression to compensate [209]. Further, an inverse correlation between decreased NO levels and increased CRH was demonstrated in preeclamptic patients compared to normal, healthy counterparts [210]. In a cohort of pregnant women exposed to phthalates, CRH was observed to be more highly expressed and correlated with the presence of gestational hypertension [211]. A separate study investigating the effects of MEHP using primary cytotrophoblasts revealed a dose-dependent increase of CRH expression and decreased cellular viability [212]. This surge in CRH upon phthalate exposure can be potentially mediated through the upregulation of TET3 [213]. In coordination with RE1-silencing transcription factor (REST), TET3 is revealed to upregulate CRH expression through modulation of DNA methylation and hydroxymethylation [214]. Moreover, increased TET3 expression has also been identified in preeclamptic patients [215]. This outlines a potential route through which phthalate exposure can epigenetically dysregulate vital pathways responsible for gestation and blood flow to be conducive to preeclampsia development.

Recently, the new toxicants of micro- and nanoplastic particles (MNPs) are rapidly being investigated for their impact on environmental and human health [216–218]. These plastic particles are defined according to their size: microplastics are 1 µm to 5 mm in size, whereas nanoplastics are 1 nm to 1 µm [219]. MNPs are found in the air, soil, food, and make their way into the body through inhalation, ingestion, and dermal contact [220]. The roles of MNPs in preeclampsia development upon exposure during pregnancy are recently being postulated [19, 221]. A study by the University of California, Los Angeles correlated that the regions with higher exposure risk to plastics through waste sites were more inhabited by the Hispanic population than any other race or ethnicity [222]. The research group went on to suggest the monitoring of microplastics and related contaminants in future research [222]. Due to their low biodegradation and persistence in the environment, MNPs are found to accumulate in several bodily tissues including reproductive organs and the placenta [219, 223, 224]. Further, MNPs were exhibited to have the capacity to cross the placental barrier and be transported to the fetus [225]. The presence of MNPs in the placenta can not only disrupt normal biological functions contributing to conditions such as preeclampsia, but the possible effects of direct fetal exposure to MNPs are still virtually unknown [226–228]. MNP exposure is described to lead to increased oxidative stress, inflammation, and disruptions in vascular health [229, 230], all of which are intimately linked to the development of preeclampsia [231, 232]. The excessive exposure of the Hispanic population to these plastic-originating toxicants may possibly provide insight on the disparate rates of preeclampsia in the Hispanic community.

Contrastingly to phthalate exposure, epigenetic research in MNP exposure and preeclampsia is still limited. Initial studies in marine life have pointed to transgenerational differences in DNA methylation with exposure to MNPs leading to altered gene expression [233]. Specifically, exposure to MNPs dysregulated DNA methylation transgenerationally and impeded the ability to reproduce [234]. This reveals the possible epigenetic impact that MNP exposure can have on pregnancy outcomes, although further mechanistic epigenetic studies bridging this environmental exposure to preeclampsia are required. Despite very limited epigenetic research linking MNP exposure to preeclampsia, research into more direct gene and protein dysregulations is emerging. In a study by Hu et al., HTR-8/sVneo cells were exposed to polystyrene (PS) MNPs to evaluate their potential effects on the placenta [235]. MNPs were discovered to migrate into the cytoplasm of the trophoblasts to increase oxidative stress, hinder invasion and migration capacities, and induce apoptosis in a dose-dependent manner [235]. Further, MNP exposure has been indicated to lead to inflammatory cytokine production, such as tumor necrosis factor alpha (TNF-α) and IL-6 through activation of the Nuclear Factor Kappa B (NF-κB) signaling pathway in mice [236, 237]. The generation of oxidative stress upon MNP exposure through the production of reactive oxygen species (ROS) may also cause DNA breaks and release ROS from nuclei and mitochondria [238]. This leads to the conclusion that MNPs can potentially be connected to preeclampsia through the generation of oxidative stress, a hallmark of preeclampsia. As plastics are found prevalently throughout our environment, the potential for human penetrance of their plasticizers and MNPs is increased. This scenario is significant as Hispanic women are highly exposed to this myriad of environmental toxicants.

## Conclusion

**Fig. 2:**
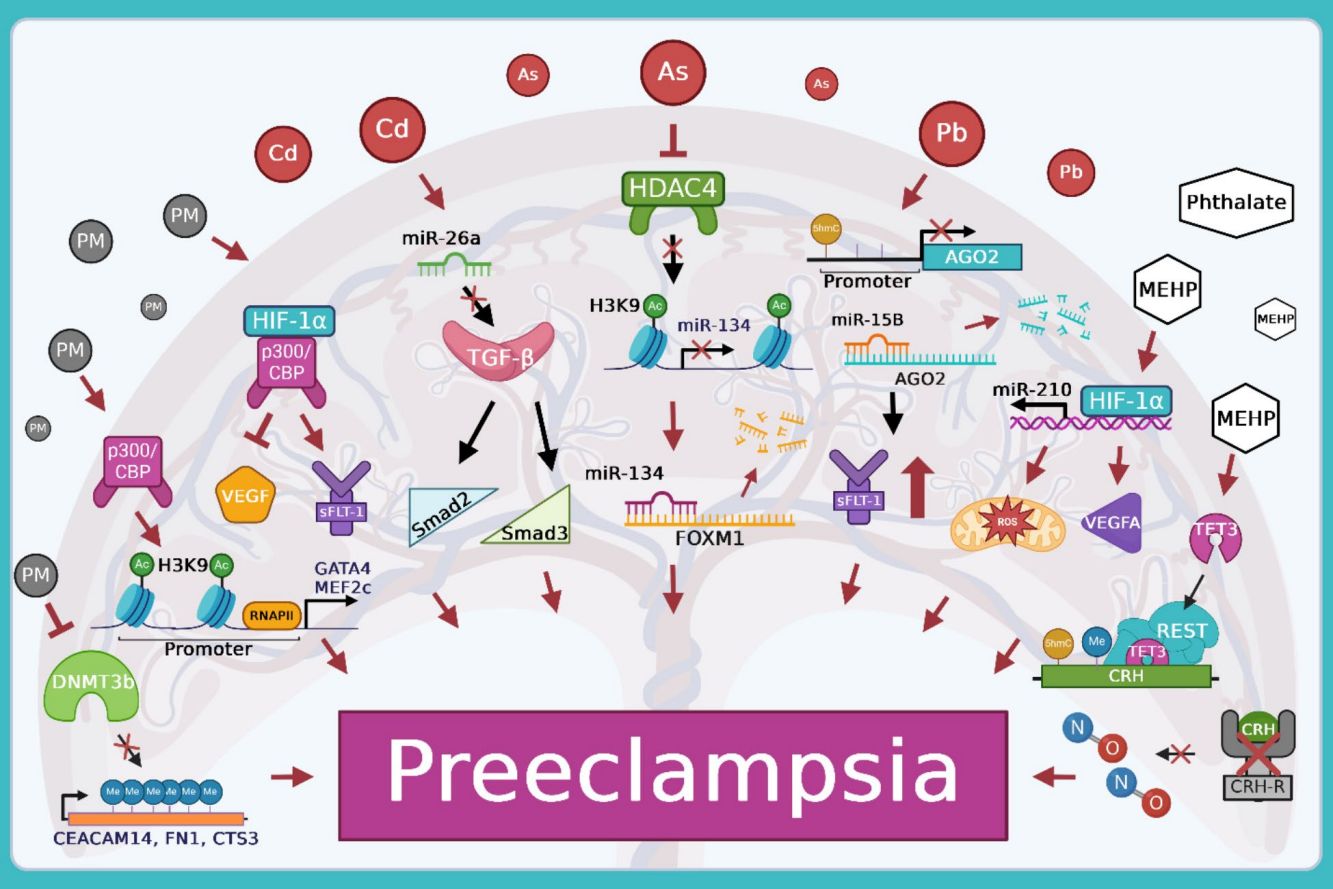
Summarizing epigenetic mechanisms disrupted by environmental toxicants leading to preeclampsia. Exposure to environmental toxicants such as air pollutants, heavy metals, and phthalates can alter epigenetic regulations within the in utero environment. This can result in modification of downstream genes and proteins expression levels that can contribute to preeclampsia. As the epigenetic mediators for MNP exposure remain unknown, they were not included. Created with BioRender

The cause for the significant contributor to maternal morbidity and mortality that is preeclampsia remains to be investigated [28]. Further, the explanation for the disproportionately high rates of preeclampsia in Hispanic women in the United States also remains elusive. To begin to shed light on this situation, this review draws together literature on preeclampsia with a Hispanic-focused lens and frames it to consider the role of epigenetics in the risk of its development (Fig. 2). We emphasized the role of different environmental toxicants such as air pollutants, heavy metals, as well as phthalates and MNPs to which Hispanic women may be disproportionately exposed as evidenced by several epidemiological studies. This racial and ethnic-oriented approach revealed several epigenetic indices through which these toxicants may lead to preeclampsia. It is necessary to recognize that exposure to these toxicants can occur simultaneously via various pathways. It is also important to note that to our knowledge, there is no literature on the proportional epigenetic risk that each toxicant may hold in the development of preeclampsia. Therefore, while further research is necessary to uncover the instigators individually, it is also relevant to consider exposure studies with a mixture of toxicants as a whole. This multifaceted approach may begin to fill in the blanks as to how Hispanic women face higher rates of preeclampsia than Non-Hispanic White women. Additionally, there are other populations such as the Non-Hispanic Black community that also have high prevalence of preeclampsia. Through comparison of metabolites or epigenetic biomarkers in non-invasive samples of various ethnicities and races living within the same region, light can be possibly shed on the linkage between different exposure levels and the epigenetic concepts. As these epigenetic dysregulations can occur before the onset of the disease itself and can be reversible, the targeting of them offers hope in potentially reversing the alterations caused by environmental toxicant exposure to aid in disease prevention. In conclusion, through the identification of racial and ethnic-specific dysregulated epigenetic mechanisms within preeclampsia, in the near future, we can set the stage for precision medicine.

## Data Availability

No datasets were generated or analyzed during the current study.
